# Supplementary material for: Pre-vaccination transcriptomic profiles of immune responders to the MUC1 peptide vaccine for colon cancer prevention
Source: Front Immunol. 2024 Oct 10;15:1437391. doi: 10.3389/fimmu.2024.1437391 (PMC11499122; doi:10.3389/fimmu.2024.1437391)
Supplement: Supplementary Table 1 — Detailed information on antibody panels used for flow cytometric analysis. [file Table1.pdf]

Supplemental Table 1

Panel 1: Innate Phenotyping

Company	Cat #	Antibody	Color
BD	560114	STAT3 (pY705)	PerCP-Cy5.5
BD	558421	anti-NF-κB p65 (pS529)	A488
BD	563931	CD152/CTLA4	BV786
Biolegend	305440	CD86	BV711
BD	743008	CD275/ICOSLG	BV650
BD	563929	CD11c	BV605
Invitrogen	L34957	Live/Dead	Amcyan
Biolegend	318340	CD56	BV510
BD	558122	CD16	PB
BD	564303	CD19	BUV737
BD	563546	CD3	BUV395
BD	335796	HLA-DR	APC Cy7
BD	557923	CD14	A700
BD	612593	Anti-ERK1/2 (pT202/pY204)	APC
Biolegend	334321	CD40	PE-Cy7
BD	563601	BCL-2	PE-CF594
BD	555388	CD11b	PE

Panel 2: MTOR Signaling

Company	Cat #	Antibody (Clone)	Color
BD	338426	CD16	PerCP-Cy5.5
BD	560048	anti-Akt1 (PKBa/Akt)	FITC
BD	564058	CD56	BV786
BD	563677	CD8	BV711
Biolegend	304135	CD45RA	BV650
Invitrogen	Q10008	CD4	Q.605
Invitrogen	L34957	Live/Dead	Amcyan
Biolegend	302242	CD19	BV510
BD	561457	pS6 (S235/S236)	V450
BD	564444	CD14	BUV737
BD	565885	Anti-ICOS (CD278)	BUV395
BD	563588	Anti-Human CD154 (CD40L)	APCeFluor780
BD	557943	CD3	A700
BD	560432	Anti-S6 pS240	A647
BD	562321	CD19	PE-CF594
BD	560684	Anti-Human CD28	PE-Cy7
BD	560285	p4E-BP1 (T36/46)	PE
