# Supplementary material for: Pre-vaccination transcriptomic profiles of immune responders to the MUC1 peptide vaccine for colon cancer prevention
Source: Front Immunol. 2024 Oct 10;15:1437391. doi: 10.3389/fimmu.2024.1437391 (PMC11499122; doi:10.3389/fimmu.2024.1437391)
Supplement: Supplementary file 2 [file DataSheet1.docx]

**Supplemental Methods:**

**Causal Modeling with MGM-FCI-MAX**

A Probabilistic Graphical Model (PGM) represents the joint distribution of variables in a dataset as a graph where each node corresponds to a variable and an edge between two nodes, A and B, corresponds to a conditional dependence between A and B given the rest of the variables in the data (1). PGM’s come in two types: directed graphical models rely on additional assumptions to infer cause and effect direction between variables, while undirected graphical models indicate only conditional dependence.

MGM-FCI-MAX is a new method to learn a directed model (2). The algorithm begins by inferring an undirected graphical model using the Mixed Graphical Models (MGM) algorithm (3) and then uses FCI-MAX to determine causal direction. MGM models categorical variables as multinomial and continuous variables as Gaussian with a mean given by a linear regression on all other variables. The full joint distribution of the model is given in Equation 1 below. Here, $x_{s}$ is the sth of p continuous $y_{j}$ is the jth of q categorical variables. $\beta_{st}$ is the linear interaction term between two continuous variables, and $\alpha_{s}$ is the continuous node potential. $\rho_{sj}$ is the edge potential function between continuous and categorical variables, and it takes on one value for each category of the variable $y_{j}$. Finally, $\phi_{rj}$ is the potential function between two categorical variables with a unique value for all combinations of categories of the variables $y_{r}$ and $y_{j}$.

$p\left( x,y;\theta\right)\propto exp\left( \sum_{s=1}^{p} \sum_{t=1}^{p} -\frac{1}{2}\beta_{st}x_{s}x_{t}+ \sum_{s=1}^{p} \alpha_{s}x_{s}+ \sum_{s=1}^{p} \sum_{j=1}^{q} \rho_{sj}\left( y_{j} \right)x_{s}+\sum_{j=1}^{q} \sum_{r=1}^{q} \phi_{rj}\left( y_{r},y_{j} \right) \right)$ (1)

The pseudolikelihood approach was used to optimize the model parameters (4). The pseudolikelihood is the product of the conditional distributions of each variable, and it is a consistent estimator of the goodness of fit of the model to the data. To ensure a sparse graph, edges are penalized via the method proposed in (3) with separate penalty parameters for each edge type: (CC = Continuous-Continuous, CD = Continuous-Discrete, DD= Discrete-Discrete) (Equation 2). Here, $\tilde{l}\left( \Theta\right)$ is the negative-log pseudolikelihood and the rest are penalty terms which ensure a sparse model.

${argmin}_{\Theta} \tilde{l}\left( \Theta\right)+\lambda_{CC}\sum_{\varphi<\omega} \left| \beta_{\varphi\omega} \right|_{1}+\lambda_{CD}\sum_{\omega,\tau} \left\| \rho_{\omega\tau} \right\|_{2}+\lambda_{DD}\sum_{\delta<\omega} \left\| \gamma_{\delta\omega} \right\|_{F}$ ($SEQ Equation \backslash* ARABIC$ $2$)

FCI-MAX is used to determine causal directions using the undirected graph as a starting point. FCI-MAX is an extension of the Fast-Causal Inference (FCI) algorithm (5), which is a sound and complete constraint-based algorithm for learning the causal structure of a set of variables in the presence of confounding variables. FCI uses conditional independence tests to rule out unlikely cause and effect relationships. FCI-MAX improves the accuracy of FCI by performing additional tests to more accurately assign orientations, especially in datasets with small sample sizes. The output of the algorithm is a graphical causal model where there are four possible edges. An edge of the form (“A --> B”) suggests that A is a cause of B and B is not a cause of A. An edge (“A <--> B”) suggests that neither A nor B is a cause of the other, that is, a confounding variable causes both. An edge (“A o--> B”) suggests that if there are no latent variables causing both A and B, then A is a cause of B. Finally, an edge of the form (“A o-o B”) suggests that both endpoints are inconclusive. In high dimensional datasets (small sample size, large number of variables) these algorithms are less accurate in inferring causal orientations as they are in inferring the presence or absence of an edge(2).

A likelihood ratio independence test (6) suitable for mixed data was used by FCI-MAX. All three sparsity parameters for MGM ($\lambda_{CC}, \lambda_{CD}, \lambda_{DD})$ were set to the default 0.2 and α = 0.1 was used for the independence test threshold for FCI-MAX. MGM-FCI-MAX was run on 100 bootstrap samples of the data, and edges were included in the final model if they appeared in at least 10% of bootstrapped samples.

**Computational Model Development and Evaluation**

LASSO logistic regression (7) was used to develop a prediction model (select genes and infer logistic regression coefficients) for a binary outcome of response defined by the clinical trial endpoint ($\geq$2-fold increase in IgG from baseline to week 12), using transcriptomic data from responders and non-responders measured two-weeks post-vaccination (Week 2 data). To develop and simultaneously evaluate model predictions, a nested leave-one-out cross validation approach was used. Iteratively, each individual sample is used as an evaluation set with the remaining samples used to learn model parameters. On each training set, a LASSO logistic regression was performed with an internal leave-one-out cross validation to choose the optimal sparsity penalty value (λ). The predictions on the single left-out sample in each round of cross validation were then used for downstream analysis.

The Receiver Operator Characteristic (ROC) curve was calculated, and predictive accuracy of the model was measured using the area under the curve (AUC) of response vs. non-response, as well as sensitivity and specificity of predicted probabilities. Feature stability was measured to ensure that models remained similar across different cross-validation iterations.

MGM-FCI-MAX was used to infer the variables directly linked to response, using clinical data (age, sex, and BMI) and those genes selected by LASSO in the week 2 transcriptomic data in at least one of the ten folds. LASSO logistic regression was used to build a predictive model of response in each cross-validation fold. All statistical analysis was performed in R.

REFERENCES

1. Koller D, Friedman, N. Probabilistic graphical models: principles and techniques: MIT press; 2009.

2. Raghu VK, Ramsey JD, Morris A, Manatakis DV, Sprites P, Chrysanthis PK, et al. Comparison of strategies for scalable causal discovery of latent variable models from mixed data. Int J Data Sci Anal. 2018;6(1):33-45.

3. Sedgewick AJ, Shi I, Donovan RM, Benos PV. Learning mixed graphical models with separate sparsity parameters and stability-based model selection. BMC Bioinformatics. 2016;17 Suppl 5:175.

4. Lee JD, Hastie TJ. Learning the Structure of Mixed Graphical Models. J Comput Graph Stat. 2015;24(1):230-53.

5. Spirtes P, Meek C, Richardson T, editors. Causal inference in the presence of latent variables and selection bias. Proceedings of the Eleventh conference on Uncertainty in artificial intelligence; 1995: Morgan Kaufmann Publishers Inc.

6. Sedgewick AJ, Buschur K, Shi I, Ramsey JD, Raghu VK, Manatakis DV, et al. Mixed graphical models for integrative causal analysis with application to chronic lung disease diagnosis and prognosis. Bioinformatics. 2019;35(7):1204-12.

7. Tibshirani R. Regression shrinkage and selection via the lasso. Journal of the Royal Statistical Society: Series B (Methodological). 1996;58(1):267-88.
